# Carbon nanotube porin diffusion in mixed composition supported lipid bilayers

**DOI:** 10.1038/s41598-020-68059-2

**Published:** 2020-07-17

**Authors:** Kylee Sullivan, Yuliang Zhang, Joseph Lopez, Mary Lowe, Aleksandr Noy

**Affiliations:** 1 Physics Department, Loyola University Maryland, Baltimore, MD 21210 USA; 2 Physical and Life Sciences Directorate, Lawrence Livermore National Laboratory, Livermore, CA 94550 USA; 3 School of Natural Sciences, University of California Merced, Merced, CA 94343 USA

**Keywords:** Biophysics, Nanoscale biophysics

## Abstract

Carbon nanotube porins (CNTPs), short pieces of carbon nanotubes capable of self-inserting into a lipid bilayer, represent a simplified model of biological membrane channels. We have used high-speed atomic force microscopy (HS-AFM) and all-atom molecular dynamics (MD) simulations to study the behavior of CNTPs in a mixed lipid membrane consisting of DOPC lipid with a variable percentage of DMPC lipid added to it. HS-AFM data reveal that the CNTPs undergo diffusive motion in the bilayer plane. Motion trajectories extracted from the HS-AFM movies indicate that CNTPs exhibit diffusion coefficient values broadly similar to values reported for membrane proteins in supported lipid bilayers. The data also indicate that increasing the percentage of DMPC leads to a marked slowing of CNTP diffusion. MD simulations reveal a CNTP-lipid assembly that diffuses in the membrane and show trends that are consistent with the experimental observations.

## Introduction

Lipid membranes play a key role in living systems by providing a structural barrier that separates cellular compartments. Bilayer fluidity in the lateral plane is a key property of lipid membranes, that allows the membrane to have sufficient flexibility to accommodate dynamic stresses, shape changes and rearrangements accompanying the cellular lifecycle^1^. 2D fluidity also underpins the membrane’s ability to incorporate a variety of receptors, porins, transporters, and pumps that mediate transport across this barrier. The ability of these proteins and protein complexes to diffuse within the bilayer plane is also integral to their function, giving this membrane environment the ability to evolve and reconfigure in response to external stimuli^2^. This functionality is also closely linked to the lipid membrane composition. Most biological membranes contain a mixture of different lipids, and small differences in lipid shape and chemical structure alter the fluidity of the membrane, which in turn affects the kinetics of protein diffusion and association in that environment^3,4^.

Our research group has developed a family of carbon nanotube porins (CNTPs), synthetic membrane nanopores based on short 1.5 nm diameter carbon nanotube scaffolds that mimic the geometry and major functionality of membrane porin proteins, which can self-insert into lipid bilayers and live cell membranes to form transmembrane pore channels with tunable permeability and ion selectivity properties^5^. We have also recently used high-speed atomic force microscopy (HS-AFM) imaging of supported lipid bilayers to demonstrate that CNTPs are mobile in the lipid membrane plane. HS-AFM is a versatile nanoscale imaging technique^6^ that is capable of visualizing real time motion of individual proteins and other large molecular objects in a variety of environments^7–9^. HS-AFM has been used to visualize myosin-V walking^10^, rotation of F_1_-ATPase^11^, dynamics of nucleosomes^12^, and dynamics of amyloid-β oligomers^13^. HS-AFM revealed an impact of cationic polymers on aggregation of β-amyloid-1–40 and amylin^14^, and fusion of peptide-based nanodisks^15^. This technique is also ideally-suitable for visualization of membrane protein motion, as demonstrated by the experiments on monitoring OmpF dynamics^16^. Our recent experiments showed that CNTP diffusion coefficients, determined from HS-AFM trajectories of CNTPs in mixed DOPC-DPPC lipid membranes on mica surfaces^17^, were of the same order of magnitude as those of membrane proteins in similar supported lipid bilayers.

We hypothesized that the overall membrane fluidity has a direct influence on the value of the lateral diffusion coefficient, *D*. To test this hypothesis, we studied CNTP diffusion in mixed DMPC-DOPC composition membranes. As the phase transition temperature of DMPC lipid (24°C)^18^ is significantly higher than that of DOPC lipid (− 17 °C)^19^, we expected that a higher DMPC content would slow down CNTP lateral diffusion. We used HS-AFM to record real-time diffusion trajectories of CNTPs in supported lipid bilayers and extract the corresponding values of *D*. The data reveal that CNTP diffusion indeed follows the expected trends. We also performed molecular dynamics (MD) simulations of CNTPs in DOPC-DMPC bilayers. MD results support our experimental findings and provide further understanding of the structure and movement of CNTPs in this environment.

## Results and discussion

**Figure 1:**
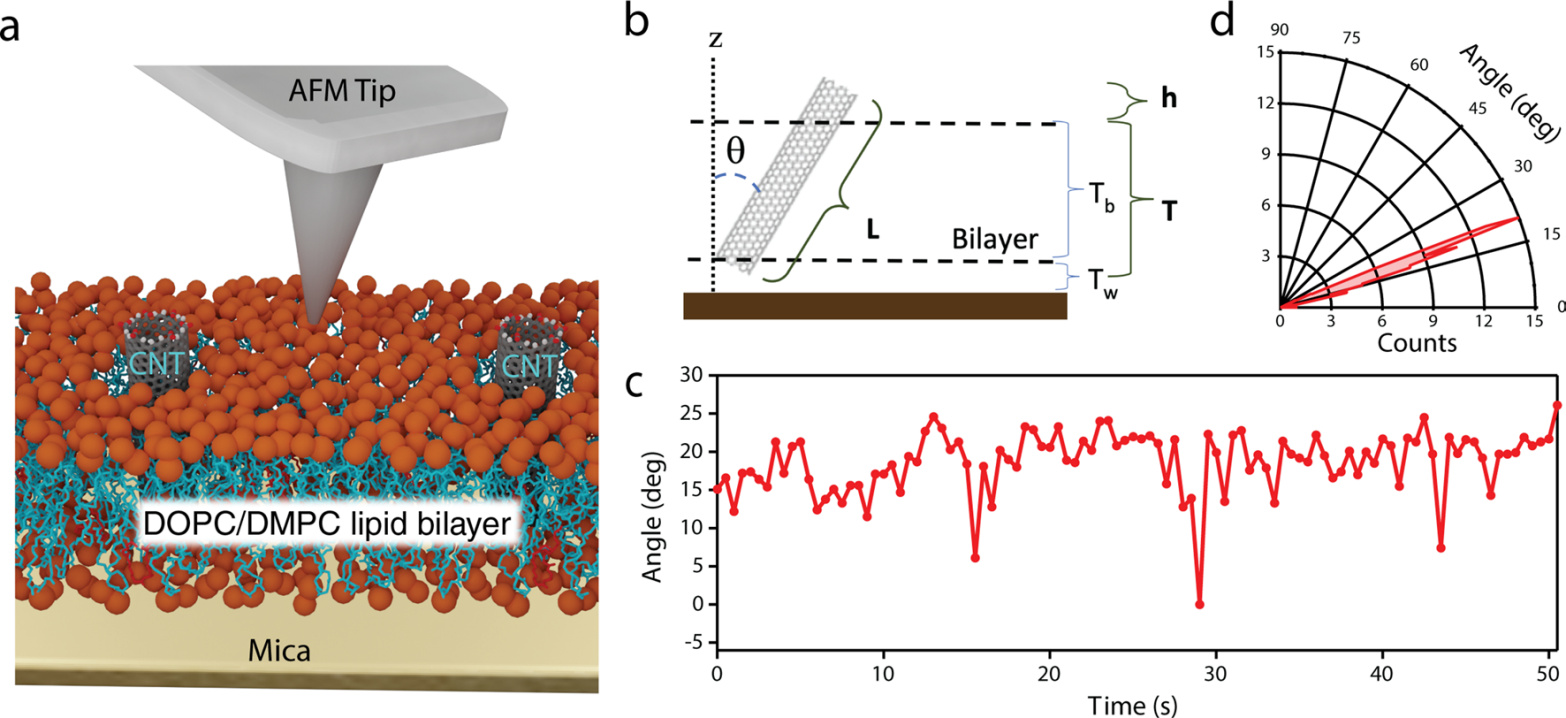
HS-AFM experimental setup and CNTP tilt angle analysis. (**a**) Schematic of the setup for HS-AFM experiments. The image shows an AFM probe on top of a lipid bilayer with inserted CNTPs. The lipid bilayer heads are depicted as orange spheres, and the corresponding lipid tails as teal lines. (**b**) Geometrical model that relates the changes in the CNTP tilt angles to the apparent CNTP height, *h*, in the AFM images. Model parameters were: bilayer thickness, *T*_*b*_ = 4.1 nm; water layer thickness, *T*_*w*_ = 1 nm; and length of CNTP, *L*, determined from the maximum height of the CNTP above the bilayer surface; (**c**) Representative tilt angle time trajectory for an individual CNTP embedded in a 50:50 DOPC-DMPC composition bilayer. (**d**) Polar plot of the histogram of the CNTP tilt angles from the time trace on the panel **c**.

**Figure 2:**
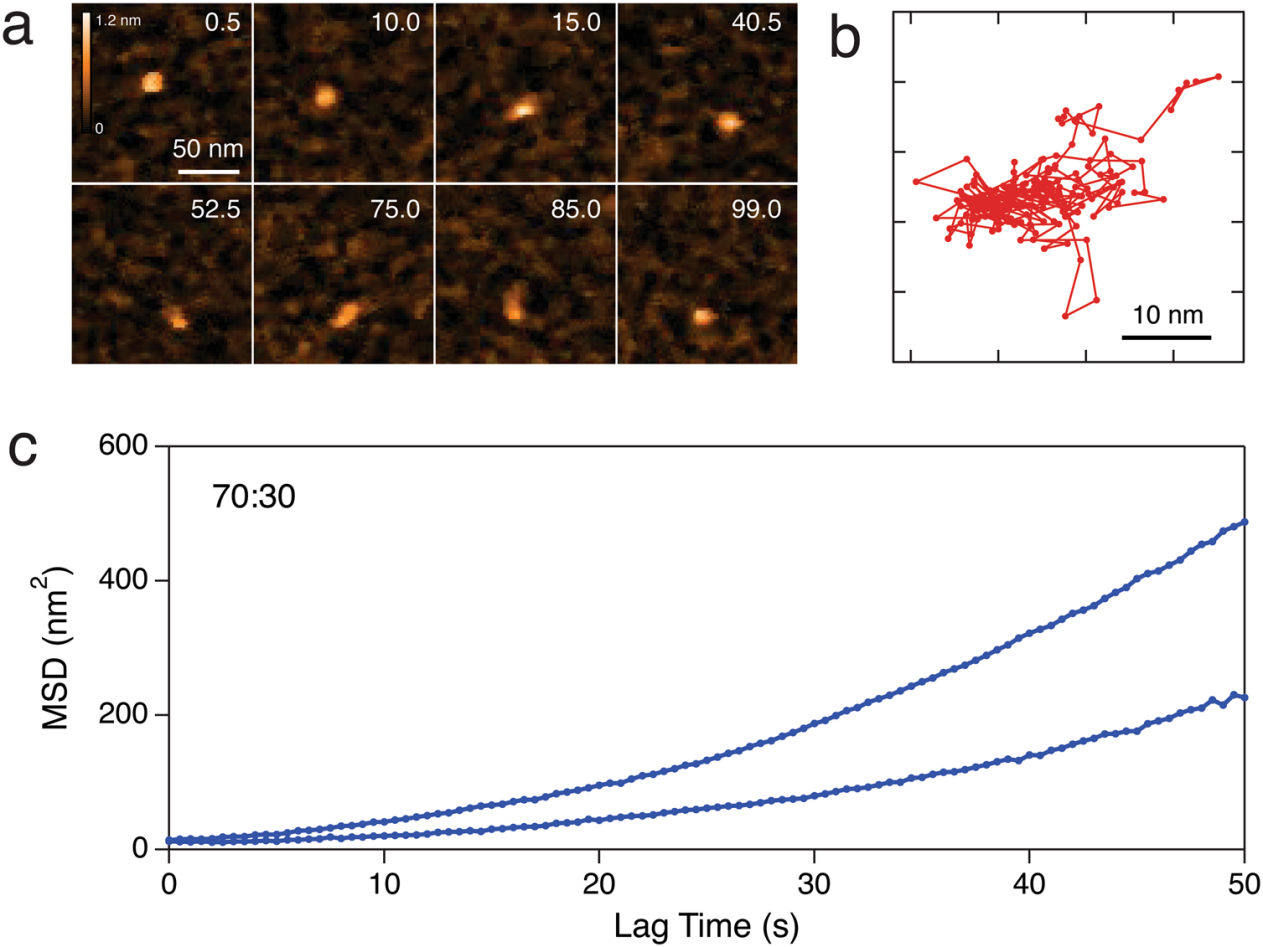
CNTP motion in supported lipid bilayers. (**a**) Representative frames (with times in seconds indicated on each image) from an HS-AFM movie showing a CNTP diffusing in a supported lipid bilayer with 80:20 DOPC-DMPC ratio (see also Supplementary Movie 2). (**b**) A representative trajectory for CNTP diffusion in the bilayer. The time step between each datapoint is 0.5 s. (**c**) Mean square displacement computed for two representative CNTP diffusion trajectories for 70:30 DOPC:DMPC ratio.

To reveal the motion of CNTPs, we used in-situ HS-AFM to image CNTPs inserted in lipid bilayers of varying DOPC-DMPC phospholipid concentrations supported on a mica sample surface (Fig. 1a). The HS-AFM images showed CNTPs as distinct 2.9 ± 0.4 nm (n = 74) high “bumps” on the lipid bilayer surface (Fig. 2a), consistent with prior observations^17^. The majority of CNTPs observed in these images did not remain stationary but, instead, moved around the bilayer in a pattern consistent with random thermally-activated Brownian diffusion (see Supplementary Movie 1). The shape of the CNTP bumps were typically asymmetric, indicating that CNTPs were tilted in the bilayer. We assessed the values CNTP tilt angles using a simple geometrical model that assumes that variations in the nanotube tilt are reflected in the fluctuations of the apparent height of the CNTP “bumps” (Fig. 1b,c). The range of tilt angles determined by this procedure (0–25 deg, see Fig. 1c,d) was slightly broader than the tilt angle range observed previously in cryo-EM experiments^5^.

We then used single particle tracking^20^ to extract individual CNTP trajectories from consecutive frames in HS-AFM movies (Fig. 2b). These trajectories were then converted to the mean square displacement (MSD) characteristics to extract the CNTP diffusion coefficient *D*. We only used the first 1/3 of the MSD trajectory to determine *D*^21^. As was also reported in previous studies, the MSD graphs (Fig. 2c) showed deviations from the linear shape expected for pure Brownian motion, suggesting the possible presence of a small flow in the supported lipid bilayer^17^. However, this effect was too small for the first 1/3 of the trajectory to require us to include a directed motion component into the fitting model.

**Figure 3:**
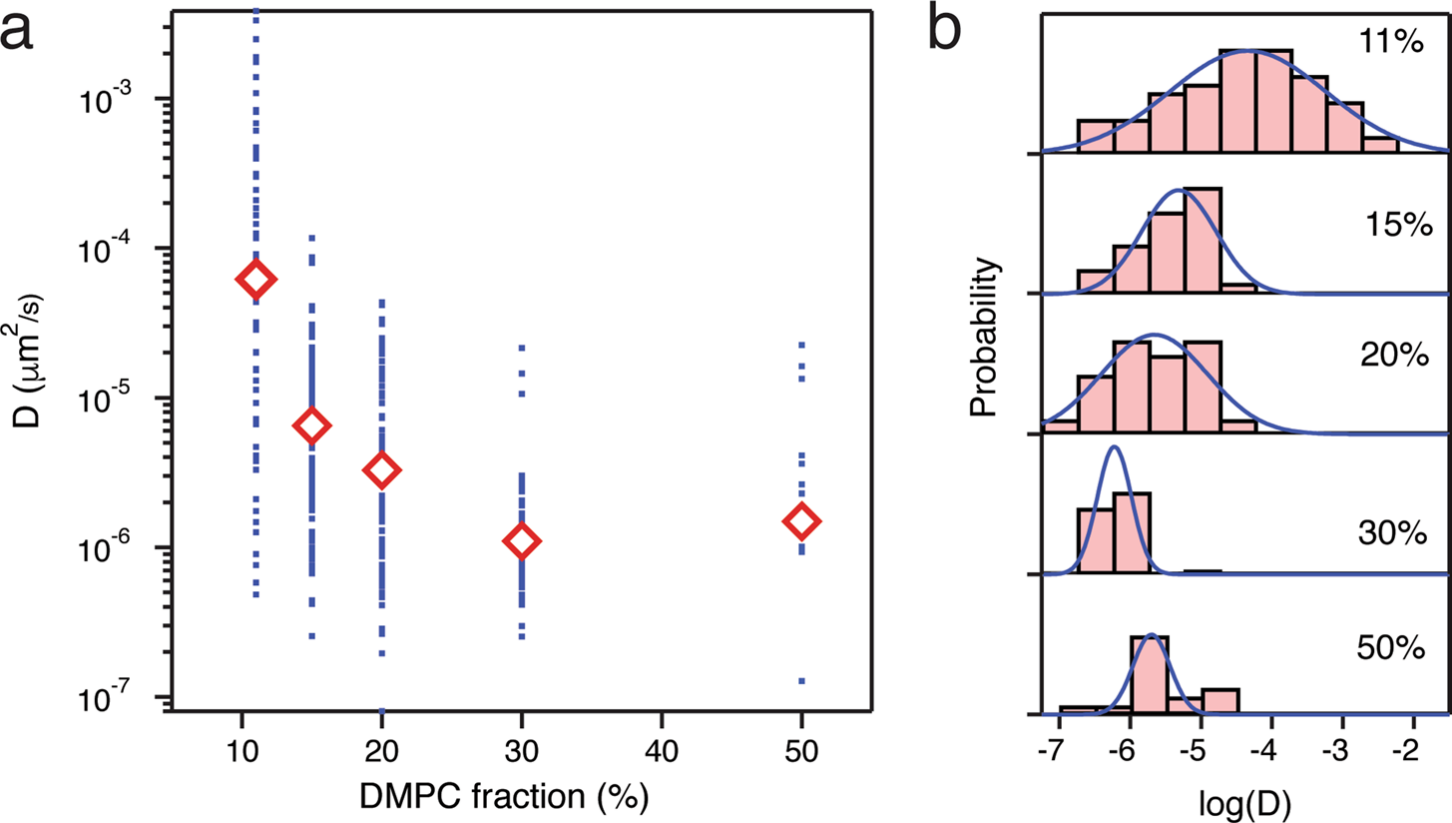
Diffusion coefficient of individual CNTPs as a function of lipid bilayer composition. (**a**) Diffusion coefficient values obtained from fitting individual CNTP trajectories (blue dots) and average values for given lipid bilayer composition (red diamonds). (**b**) Distribution of individual diffusion coefficient values for a bilayer with 20% DMPC fraction. Blue solid lines represent best fits to log-normal distributions.

For each lipid composition, *D* values for individual CNTPs span a relatively wide range (Fig. 3a), reflecting the inhomogeneous distribution of CNTP lengths and, possibly, different degrees of CNTP end interactions with the mica surface supporting the bilayer. Overall, the values generally follow a log-normal distribution (Fig. 3b) peaking at a defined range. The averages of these distributions (Fig. 3a) show a clear trend where the diffusion becomes slower with an increased fraction of DMPC lipid and the corresponding decrease in bilayer fluidity.

Measured CNTP diffusion coefficient values were for the most part smaller than 0.001 μm^2^/s, which is lower than the values reported for membrane proteins in free cell membranes (0.005–0.35 μm^2^/s)^22,23^ with only some of the CNTPs embedded in a 89:11 DOPC-DMPC composition of the lipid bilayer reaching D values comparable to those of the membrane proteins. Typical reported *D* values for membrane proteins range from 0.4 μm^2^/s for rhodopsin^24^, 0.5–8 μm^2^/s for proteins in a DMPC bilayers^25^, and 0.03–0.08 μm^2^/s for peptides in POPC vesicles^26^. Notably, low values of *D* were observed in two types of systems: *D* = 10^–4^ μm^2^/s for fibronectin, a protein that interacts with the extracellular matrix^24^, and *D* = 10^–5^ μm^2^/s for ATP synthase rotors in a POPC membrane supported on mica^26^. In both of these systems the protein diffusing in the lipid membrane interacts with the underlying surface, which unsurprisingly slows down the protein diffusion. The presence of the underlying mica surface, which is unavoidable in the HS-AFM experiments, represents one of the major limitations of our approach. Furthermore small-angle X-ray scattering (SAXS) experiments^27^ showed that insertion of the CNTP into a DOPC bilayer causes the thickness of the bilayer to become thinner by about 0.35 nm, which could potentially affect *D* values.

**Figure 4:**
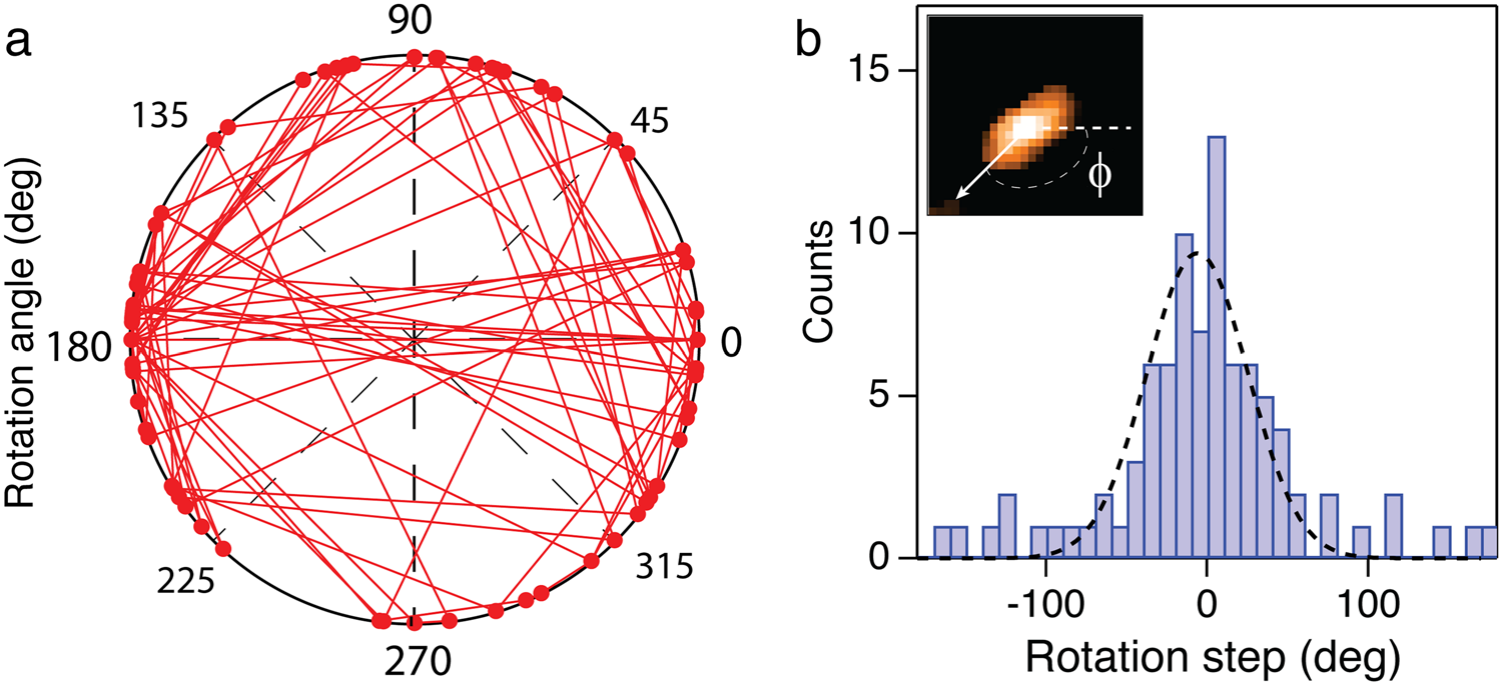
Rotational diffusion of CNTPs. (**a**). Trajectory of the rotational diffusion of a CNTP in the lipid bilayer with 50:50 DOPC:DMPC composition. (**b**). Histogram of the values of the angle changes at each step in the trajectory. Dashed line indicates a Gaussian fit to the histogram. Inset shows an example of rotational angle being defined by the shape asymmetry of the CNTP “bump” in the AFM image.

HS-AFM images also provide an interesting possibility to track rotation of the CNTP from the asymmetry of the CNTP “bumps”. This analysis allowed us to capture CNTP rotational trajectories (Fig. 4a) and characterize CNTP rotational steps (Fig. 4b). However, the time resolution of the HS-AFM imaging was insufficient to track the rotation with enough accuracy to capture the details of the rotational dynamics or to measure cumulative rotation angle of the trajectory. Thus, we were unable to determine a rotational diffusion coefficient of the CNTP in the lipid bilayers.

**Figure 5:**
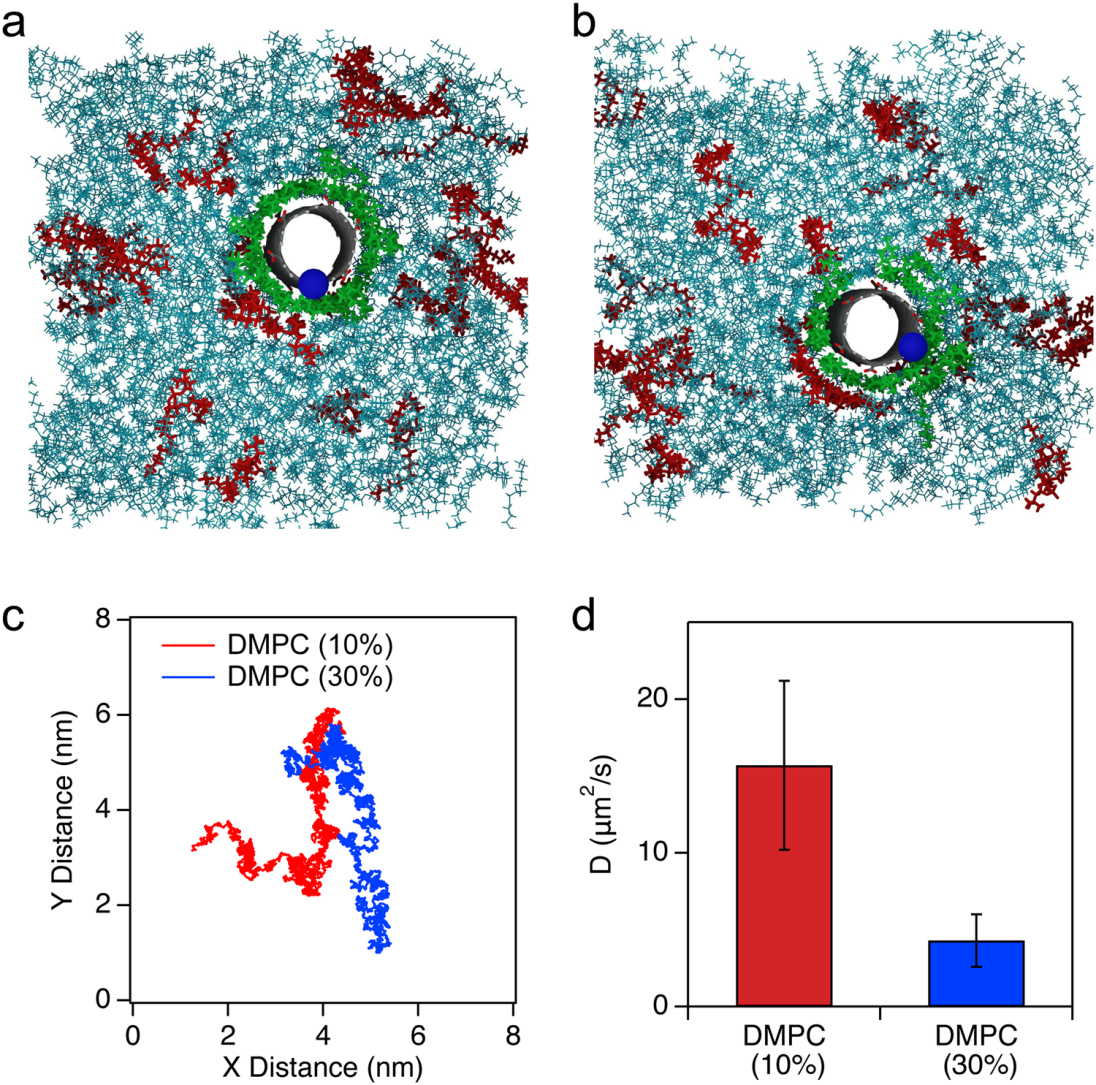
MD simulations of the configuration and the dynamics of CNTPs in lipid bilayers with different DOPC-DMPC compositions. (**a**) Snapshot of the CNTP-bilayer system at 100 ns with 90:10 DOPC-DMPC composition. (**b**) Snapshot of the same CNTP-bilayer system at 200 ns. The blue dot represents a carbon atom chosen as an orientation indicator on the rim of the CNTP. The lipid annulus surrounding the CNTP is colored in green. DOPC and DMPC are shown as cyan and red sticks, respectively. (**c**) Trajectories for CNTP lateral diffusion for two DOPC-DMPC ratios. (**d**) Comparison of diffusion coefficient values for two lipid compositions.

To gain more insight about the configuration and dynamics of the CNTP in lipid bilayers, we performed 200 ns molecular dynamics (MD) simulations of short segments of carbon nanotubes inserted in a lipid bilayer (Fig. 5a,b). The simulations indicate that CNTPs can move laterally and rotate freely inside the bilayer. We monitored the orientation changes of a CNTP by tracking the location of one carbon atom on the CNTP rim (marked as a blue ball on Fig. 5). The rotation is relatively slow: over 100 ns, the position of the carbon rotates from the 6 o’clock (Fig. 5a) to the 4 o’clock position (Fig. 5b, see also Supplementary Movie 3). Interestingly, we observed a tightly-bound annular lipid ring around the nanotube (green structure in Fig. 5) that includes both DOPC and DMPC molecules. The phenomenon is similar to the behavior reported in a recent study^28^. We were not able to discern any preference for a particular lipid adsorption to the CNTP surface, which likely reflects the non-specific nature of the hydrophobic interactions between the lipid tail and the CNTP. This annular ring is very stable: it did not change as the nanotube diffused and rotated in the bilayer and the ring did not dissociate over the 100 ns timescale of the simulation production run (Fig. 5b, see also Supplementary Movie 3). We note that this ring should also effectively increase the radius of the diffusing structure in the membrane.

We further analyzed the lateral diffusion behavior for two DOPC-DMPC (90:10 and 70:30) compositions (Fig. 5c,d). Although it is impossible to make a direct comparison between simulations, which model an unsupported lipid bilayer, and experiments, which probe a supported lipid bilayer, we found that the calculated CNTP diffusion coefficients (Fig. 5d), using a directed diffusion model (Supplementary Fig. S1), followed the same trend with lipid composition observed with HS-AFM data, see Fig. 3. In the simulations *D* varied from 21.7 μm^2^/s (90:10 DOPC-DMPC) to 3.1 μm^2^/s (70:30 DOPC-DMPC). This range is smaller than the three orders of magnitude variations observed in HS-AFM data, again underscoring the complexity of the interactions in the experiments. We also did not observe any significant aggregation of DMPC molecules in the simulations, indicating that the bilayer represents a true mixture. Perhaps longer simulations of this system will reveal more information about its temporal evolution; however, these calculations are computationally expensive and would likely require coarse-grained simulations to obtain significantly longer time trajectories.

## Conclusions

Here, we used an HS-AFM to capture and analyze the lateral motion trajectories of CNTPs in supported mixed lipid bilayers at varying DOPC-DMPC ratios. An increase in the DMPC component, which has a significantly higher phase transition temperature than the DOPC lipid, increases the overall lipid bilayer viscosity, leading to a corresponding slowdown in the CNTP lateral motion in the bilayer plane. MD simulations revealed several interesting features of the CNTP-lipid system, including the existence of a tightly bound annular lipid ring around the CNTP; the ring diffuses with the CNTP. This study confirms that CNTPs mimic the major features of the diffusive movement of biological pores in lipid membranes and shows how the increase in bilayer viscosity leads to a corresponding slowdown in protein motion. It should be possible to extend this approach to studies of other membrane protein dynamics in supported lipid bilayers. We note that those studies, however, will need to be mindful of the challenge of unambiguous visualization of the membrane components, especially in systems that incorporate smaller proteins, such as antimicrobial peptides. Another challenge that could complicate these studies would be microscopic phase separation of the lipid matrix that could lead to complicated pore dynamics in the membrane.

## Methods

### Carbon nanotube porin synthesis and incorporation into liposomes

The 1.5 nm diameter carbon nanotubes were sourced from Carbon Solutions, Inc. (P2-SWNT). 1,2-Dioleoyl-sn-glycero-3-phosphocholine lipid (DOPC) and 1,2-dimyristoyl-sn-glycero-3-phosphocholine lipid (DMPC) were purchased from Avanti Polar Lipids. The CNTPs prepared and purified by methods detailed in a recently published detailed protocol^29^. The rehydration of CNTPs in 20 mM HEPES buffer (pH 7.8), and incorporation of CNTPs into DOPC-DMPC liposomes followed the protocols described in our previous work^17^. For liposome preparations, mass ratios of DOPC-DMPC were varied as: 50:50, 70:30, 80:20, 85:15, 89:11. All liposomes were used within two days of preparation.

### Substrate preparation and liposome fusion

For HS-AFM imaging, a mica disc with a 1.5 mm diameter was glued onto the glass rod of the sample stage. The mica surface was freshly cleaved prior to sample deposition. Lipid bilayers were formed on the mica surface using a vesicle fusion technique. Briefly, 4 µL of liposome solution was deposited on the surface and incubated for 30 min at room temperature in a humidity chamber to reduce water loss due to evaporation. After incubation, the free liposomes were rinsed away with 20 μl of 10 mM HEPES buffer (pH 7.8), two times, and stored in the humidity chamber. To confirm the formation of a single lipid bilayer on the mica substrate, 0.5% Texas Red 1,2-Dihexadecanoyl-sn-Glycero-3-Phosphoethanolamine (TR-DHPE) was incorporated into a lipid mixture in order to observe lipid spreading under a fluorescence microscope. HS-AFM imaging to did not reveal any evidence of phase separation of DOPC and DMPC (see also Supplementary Fig. S2).

To confirm the insertion of CNTPs into the supported lipid bilayer, a 70:30 DOPC-DMPC sample hydrated with CNTPs was scanned with a conventional AFM. The images showed circular features with some asymmetry. Heights of 74 CNTP features yielded an average protrusion height of 2.9 ± 0.4 nm above the bilayer (see also Supplementary Fig. S3).

### HS-AFM imaging and data processing

HS-AFM images of CNTPs were acquired in tapping mode at room temperature using an HS-AFM (RIBM, Japan) equipped with ultra-short AFM cantilevers with high-density carbon/diamond-like carbon (HDC/DLC) tips (USC-F1.2-k0.15, NanoWorld, tip radius < 10 nm). The details were described in a previous publication^17^. The HS-AFM fluid cell was filled with 120 μl of 20 mM HEPES buffer. In a typical experiment, 128 pixel × 128 pixel images were collected from a 200 × 200 nm area and scanned at a rate of 0.5 s, 0.3 s, or 0.2 s per frame. The deflection sensitivity of AFM tip was of 0.1 V/nm. The free amplitude was about 20 Å and 90% of the free amplitude was chosen as imaging setpoint. Raw HS-AFM movie data were converted to ImageJ stacks. CNTP motion trajectories were extracted using the TrackMate ImageJ plugin (https://imagej.net/TrackMate). For 89:11 DOPC-DMPC, fast CNTPs were tracked manually. When necessary, mechanical drift in HS-AFM movies was corrected using an ImageJ macro developed by N. M. Schneider (https://github.com/NMSchneider/fixTranslation-Macro-for-ImageJ). Diffusion coefficients were computed from mean-square displacement values using a custom IgorPro 6 (Wave-Metrics, Lake Oswego, OR, USA) script.

### Molecular dynamics (MD) simulations

MD simulations were performed with NAMD 2.12b1^30^ using the CHARMM36^31,32^, CGenFF 3.1 force fields^33^, and TIP3P water model^34^. Initial configurations consisted of a carbon nanotube embedded in a lipid bilayer composed of DOPC and DMPC lipid molecules at two ratios: 70:30 and 90:10. The CNTPs were generated using VMD 1.9.3^35^ with the Nanotube Builder 1.5 plugin. The CNTP structure matched the diameter of the carbon nanotubes used in the HS-AFM experiments and consisted of a (14, 7) single-walled carbon nanotube with a diameter of 1.5 nm and a length of 5.5 nm. To properly reflect the end chemistry of CNTPs, which always have COO– groups formed during sonication-cutting, the CNTP was functionalized with 6 carboxylate groups at each end. The VMD Molefacture plugin was used to generate these structures. The other carbons were terminated with aromatic hydrogens at each end. Wall carbons were given a charge of 0e. Terminal carbons were given a charge of − 0.115e, and hydrogens bonded to the terminal carbons were given a charge of 0.115e^36^. For the carboxylate groups, the carbon was given a charge of 0.34e and the oxygens a charge of − 0.67e, in accordance with C-terminus (CTER) in the CHARMM36 topology file. The total charge of the CNTP was − 12e. The configurations of the DOPC-DMPC bilayers containing 226 (70:30) or 224 (90:10) lipid molecules, were generated using the CHARMM-GUI Membrane Builder^37–39^. Subsequently, the initial configuration was constructed by placing the functionalized CNTP into this bilayer in a perpendicular conformation, and then solvated in an orthorhombic box (9.4 nm × 9.4 nm × 8.4 nm) with 13,103 (70:30) or 14,313 (90:10) TIP3P water molecules. Potassium and chloride ions were used to neutralize the system and mimic the physiological salt condition of 150 mM KCl (49 K^+^, 37 Cl^-^ for 70:30; 52 K^+^, 40 Cl^-^ for 90:10).

After 4,000 steps of conjugate gradients energy minimization, the CNTP system was equilibrated for 200 ns in an isothermal-isobaric (NPT) ensemble with an integration time step of 2 fs. The latter 100 ns was used as the production run. The Langevin temperature coupling method with a friction coefficient of 1 ps^-1^ was applied to maintain the temperature at 300 K. The system pressure was maintained at 1 atm via the Nose − Hoover Langevin piston method^40^. A cut-off distance of 12 Å was used to deal with short range electrostatic and van der Waals interactions. Long range electrostatic interactions were computed using the particle mesh Ewald (PME) method^41^. The area per lipid for the 90:10 mixture was 65 Å^2^; this value is comparable to neutron and X-ray scattering data at 303 K for DOPC (67.4 Å^2^)^42^ and DMPC (59.9 Å^2^)^43^.

The location of the center-of-mass of the CNTP was calculated in each frame using the PBCTools plugin for VMD and Tcl scripts, and the diffusion coefficient was computed in Matlab based on codes from https://tinevez.github.io/msdanalyzer/.

## Supplementary information

Supplementary movie S1

Supplementary movie S2

Supplementary movie S3

Supplementary movie S4

Supplementary movie S5

Supplementary movie S6

Supplementary information
