# Supplementary material for: Carbon nanotube porin diffusion in mixed composition supported lipid bilayers
Source: Sci Rep. 2020 Jul 17;10:11908. doi: 10.1038/s41598-020-68059-2 (PMC7368039; doi:10.1038/s41598-020-68059-2)
Supplement: Supplementary file 7 — Supplementary information [file 41598_2020_68059_MOESM7_ESM.pdf]

Supplementary Information

Carbon Nanotube Porin Diffusion in Mixed Composition Supported Lipid Bilayers

Kylee Sullivan,[1] Yuliang Zhang,[2] Joseph Lopez,[1] Mary Lowe,[1,*] and Aleksandr Noy[2,3,*]

[1]Physics Department, Loyola University Maryland, Baltimore, MD 21210, USA

[2]Physical and Life Sciences Directorate, Lawrence Livermore National Laboratory, Livermore, CA 94550, USA

[3]School of Natural Sciences, University of California Merced, Merced, CA 94343, USA

Corresponding authors: Mary Lowe and Aleksandr Noy

Phone:

E-mail: (mlowe@loyola.edu) and AN (noy1@llnl.gov).

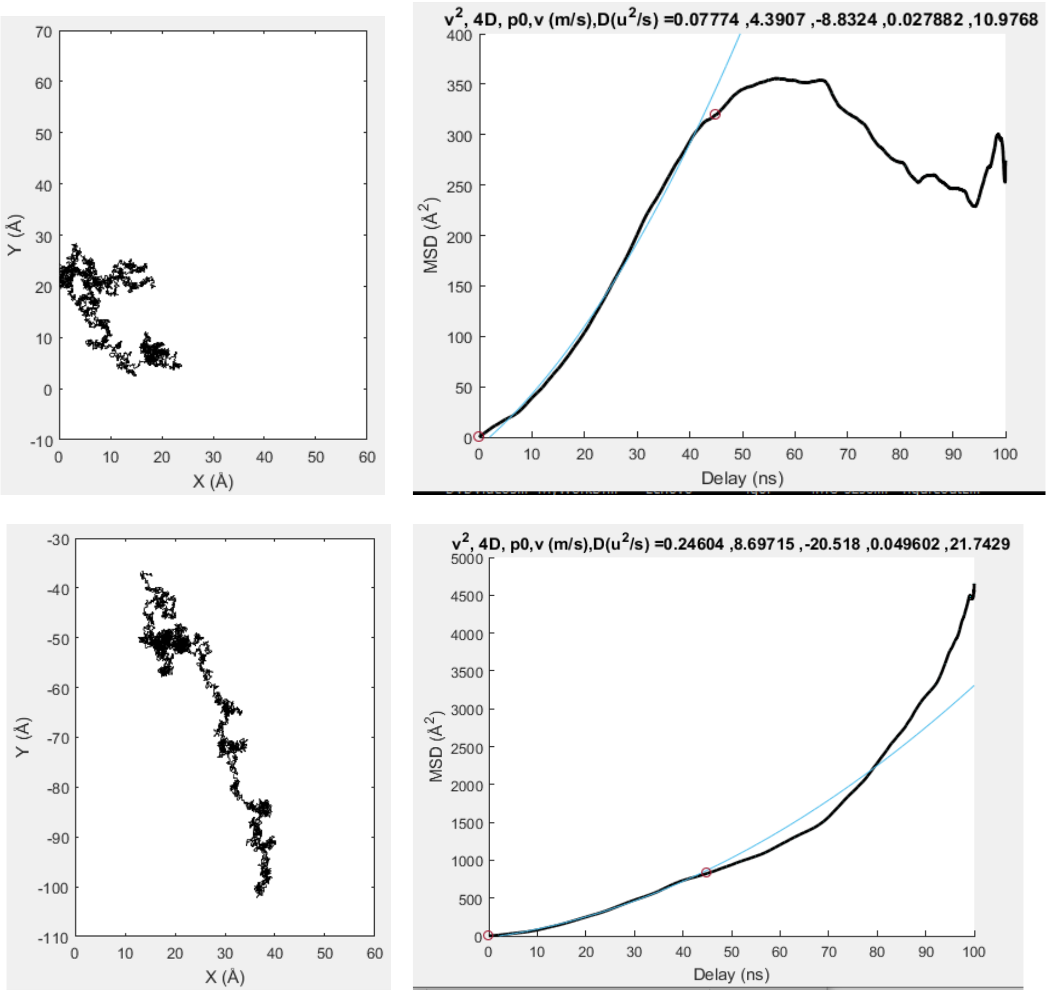

Supplementary Figure S1: Trajectories of the CNTP in two MD simulations for the 90:10 DOPC-DMPC mixture. A directed diffusion model was used to compute the diffusion coefficient for the first 45% (top, D = 11 u^2/s) of the points or 50% (bottom, D = 21.7 u^2/s).

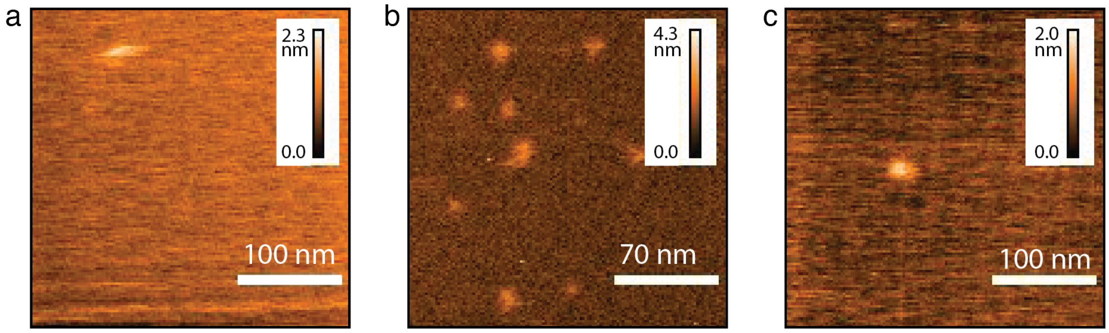

Supplementary Figure S2. Representative HS-AFM images showing the background and CNTPs for three concentration ratios of DOPC-DMPC. (**a**) 88:12, (**b**) 70:30, (**c**) 50:50. In all cases, there was no evidence of phase separation between DOPC and DMPC. The corresponding movies are given as Movie 4, Movie 5, and Movie 6, respectively.

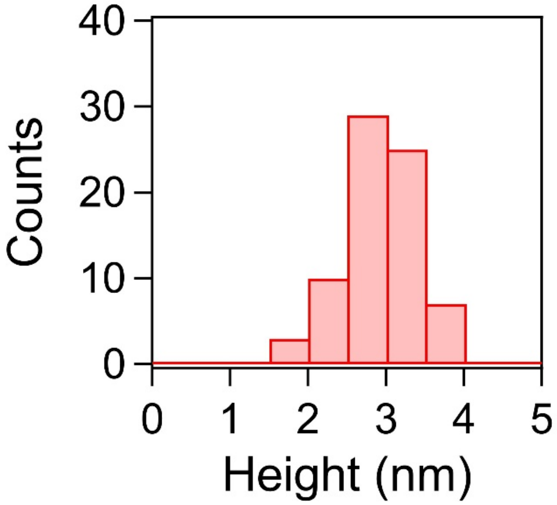

Supplementary Figure S3. Histogram of the heights of 74 features in conventional AFM images for a 70:30 DOPC:DMPC bilayer with CNTPs. The average height is 2.9 ± 0.4 nm above the surface.

Movie captions.

Movie 1: High-speed AFM movie of CNTP diffusion in supported lipid bilayer.
Filename: HS_AFM_Movie1.avi
High-speed AFM movie showing diffusion of CNTPs in a lipid bilayer of the composition of 85:15 DOPC-DMPC on mica. Frame dimensions: 300x300 nm. Imaging rate: 2 frames per second. Movie duration: 40 seconds.

Movie 2: High-speed AFM movie of CNTP diffusion in supported lipid bilayer.
Filename: HS_AFM_Movie2.avi
High-speed AFM movie showing diffusion of CNTPs in a lipid bilayer of the composition of 80:20 DOPC-DMPC on mica. Frame dimensions: 300x300 nm. Imaging rate: 2 frames per second. Movie duration: 100 seconds.

Movie 3: MD simulation of CNTP diffusion in a mixed lipid bilayer.
Filename: MD_Movie3.mov
A movie of a molecular dynamics simulation of a CNTP embedded in a mixed composition (90:10 DOPC-DMPC) lipid bilayer. DOPC and DMPC are shown as cyan and red sticks, respectively. The ring of lipids closest to the CNTP is colored in green. The blue dot represents a carbon atom on the rim of a CNTP chosen to help track CNTP rotation. Movie duration: 100 ns.

**Movie 4. High-speed AFM movie of CNTP diffusion in supported lipid bilayer. **
Filename: HS_AFM_Movie4.avi
DOPC:DMPC ratio was 88:12. Movie dimension and scale as indicated on the Figure S2a. Imaging rate: 2 frames per second.

Movie 5. High-speed AFM movie of CNTP diffusion in supported lipid bilayer.
Filename: HS_AFM_Movie5.avi
DOPC:DMPC ratio was 70:30. Movie dimension and scale as indicated on the Figure S2b. Imaging rate: 2 frames per second.

Movie 6. High-speed AFM movie of CNTP diffusion in supported lipid bilayer.
Filename: HS_AFM_Movie6.avi
DOPC:DMPC ratio was 50:50. Movie dimension and scale as indicated on the Figure S2c. Imaging rate: 2 frames per second.
